# Far-field subwavelength imaging with near-field resonant metalens scanning at microwave frequencies

**DOI:** 10.1038/srep11131

**Published:** 2015-06-08

**Authors:** Ren Wang, Bing-Zhong Wang, Zhi-Shuang Gong, Xiao Ding

**Affiliations:** 1 Institute of Applied Physics, University of Electronic Science and Technology of China, Chengdu 610054, China

## Abstract

A method for far-field subwavelength imaging at microwave frequencies using near-field resonant metalens scanning is proposed. The resonant metalens is composed of switchable split-ring resonators (SRRs). The on-SRR has a strong magnetic coupling ability and can convert evanescent waves into propagating waves using the localized resonant modes. In contrast, the off-SRR cannot achieve an effective conversion. By changing the switch status of each cell, we can obtain position information regarding the subwavelength source targets from the far field. Because the spatial response and Green’s function do not need to be measured and evaluated and only a narrow frequency band is required for the entire imaging process, this method is convenient and adaptable to various environment. This method can be used for many applications, such as subwavelength imaging, detection, and electromagnetic monitoring, in both free space and complex environments.

Because of the exponential decay of evanescent waves, which carry high spatial frequency components that contain subwavelength information, the resolution of conventional imaging systems is limited by the diffraction limit^1^. To overcome the diffraction limit, several methods have been proposed in recent years. The near-field scanning technique^2^,^3^,^4^,^5^, stimulated emission depletion microscopy^6^,^7^, stochastic optical reconstruction microscopy^8^,^9^,^10^, microspheres technique^11^,^12^,^13^,^14^, super-oscillatory lens optical microscopy^15^,^16^, perfect lenses^17^,^18^, and hyperlenses^19^,^20^ have been presented and realized. In 2003, evanescent waves were shown to be significantly enhanced across a silver slab and were demonstrated in optical experiments^21^,^22^. Subsequently, the evanescent electromagnetic wave enhancement was also observed in microwave metamaterials with negative permittivity and permeability^23^,^24^.

Evanescent wave enhancement can only be used for subwavelength imaging in the near field because the evanescent waves away from the near-field superlenses will continue to decay. To realize far-field subwavelength imaging, a silver superlens with nanoscale corrugations on its top surface was proposed to enhance the evanescent wave and to convert them into the propagating wave^25^,^26^,^27^. Moreover, select resonant structures, such as surface plasmons^28^,^29^, metallic cylinders arrays^30^,^31^,^32^, and split-ring resonators (SRRs)^33^,^34^,^35^,^36^, were used to convert the evanescent wave into a propagating wave and allow the subwavelength information to be received by the receivers in the far field. To reconstruct the target image in the far field at microwave frequencies, it is generally necessary and important to obtain the Green’s function between the targets and receivers. However, the Green’s function is difficult and time-consuming to obtain in a complex environment. When the environment changes even minimally, the spatial response and Green’s function must be remeasured and recalculated due to the environmental sensitivity of the subwavelength imaging.

In this study, a method for far-field subwavelength imaging at microwave frequencies using near-field resonant metalens scanning is proposed. The resonant metalens is composed of a planar SRR array, and each SRR cell has a switch in the middle of the metal split ring. An SRR cell with a switch that is on (on-SRR) has a strong magnetic coupling ability and can convert the evanescent wave into a propagating wave using the localized resonant modes. In contrast, the off-SRR cannot achieve effective conversion. By changing the switch status of each cell, we can obtain the position information of subwavelength source targets from the far field. Throughout the entire imaging process, the Green’s function does not to be considered and only a narrow frequency band is required. Therefore, this method is convenient and environmentally adaptable. This method can be used for subwavelength imaging, detection, and monitoring in both free space and complex environments.

## Results

### Subwavelength imaging in free space

**Figure 1:**
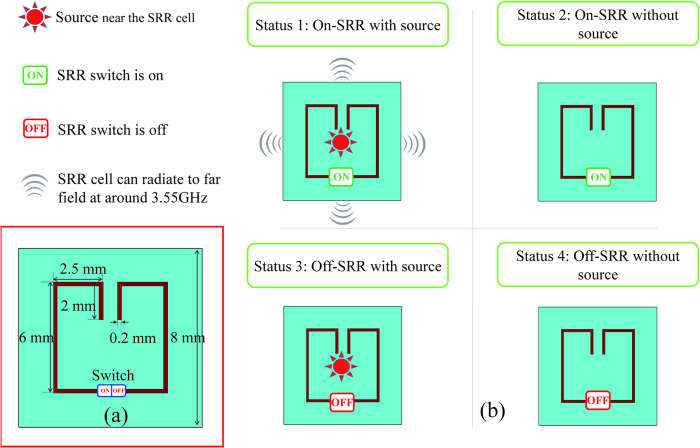
The SRR cell with a switch. (**a**) Structure of the SRR with a resonant frequency of 3.55 GHz. (**b**) The four statuses of the switchable SRR. When the subwavelength source is within the coupling scope of the on-SRR, the SRR will transmit the subwavelength information to the far field via localized mode resonance at 3.55 GHz. The off-SRR cannot achieve the effective evanescent-propagating conversion.

SRR is well adapted to realize a localized mode metalens, and its resonant frequency can be easily controlled by changing the length of the metal split ring^36^. In this study, an SRR with a resonant frequency of 3.55 GHz is designed, and its structure is shown in Fig. 1(a). The SRR cell has a switch in the middle of the metal split ring, which is printed on a dielectric substrate. The substrate used in this study has a thickness of 0.5 mm and a relative dielectric constant of 4.6. Figure 1(b) shows the four statuses of the switchable SRR. When the subwavelength source is within the coupling scope of the on-SRR, the SRR will transmit the subwavelength information to the far field via a localized mode resonance at 3.55 GHz. The strong magnetic coupling scope of the SRR cell is near the top and bottom sides. The off-SRR cannot achieve effective evanescent-propagating conversion. When a subwavelength source is with the off-SRR, no radiation at 3.55 GHz can be received in the far field because the fundamental resonance frequency of the subwavelength source is much higher than that of the SRR. As a consequence, the resonant metalens composed of switchable SRRs can be used for far-field subwavelength imaging by setting the switch status of each cell. Select imaging examples are given in the following sections.

**Figure 2:**
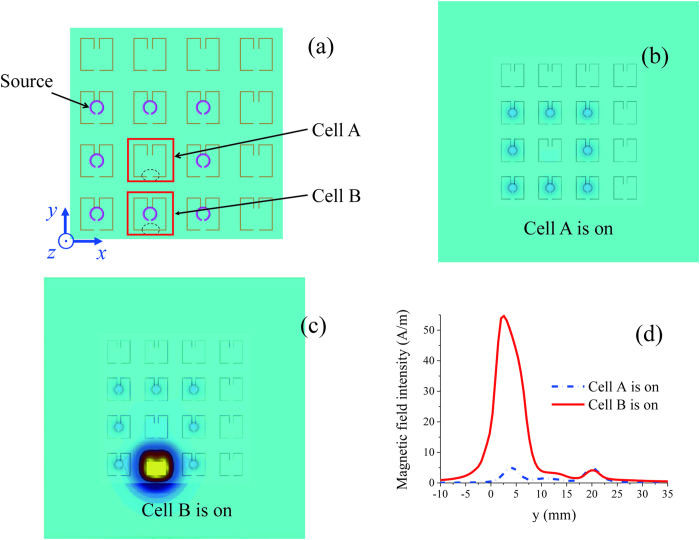
The magnetic field pattern in the plane near and below the SRR metalens at 3.55 GHz. (**a**) Structure of the SRR metalens with eight small loop sources. The metalens and small loops are located in the planes of z = 0 and z = 0.5 mm. When Cell A or Cell B is turned on, the magnetic field patterns in the plane of z = −2 mm at 3.55 GHz are shown in (**b**) and (**c**), respectively. The magnetic field intensities corresponding to (**b**) and (**c**) at the line of x = 12 mm are shown in (**d**). The line of x = 12 mm is through the centres of Cell A and B.

A metalens composed of 4 × 4 switchable SRRs (Fig. 1(a)) is used to demonstrate the far-field subwavelength imaging. The structure of the SRR metalens with eight small loop sources is shown in Fig. 2(a). The metalens and loop sources are located in the z = 0 and z = 0.5 mm planes, respectively. The eight small loops are excited simultaneously in the 3–4 GHz band to simulate a subwavelength squared target of 16 mm by 16 mm. The fundamental mode of the small loops is at 20 GHz, which is well beyond the excitation frequencies and the fundamental resonance frequency of the on-SRR.

In the imaging process, only one cell of the metalens is turned on at given time. Cell A (without a loop source above) and Cell B (with a loop source above) provide two examples to show the different excited magnetic field pattern distributions. When Cell A of the metalens is turned on, the other cells except Cell A are turned off; when Cell B is turned on, the other cells except Cell B are turned off. Below the SRR metalens and in the plane of z = −2 mm, the magnetic field patterns of Cell A and Cell B at 3.55 GHz are shown in Fig. 2(b,c), respectively. The magnetic field intensities corresponding to Fig. 2(b,c) at the line of x = 12 mm are shown in Fig. 2(d). The line of x = 12 mm is through the centres of Cells A and B. From Fig. 2, we can see that when the cell without a source above it is on, the surface magnetic field is low, and when the cell with a source above it is on, its surface magnetic field is much higher than the others. Moreover, the high magnetic field is limited to the scope of the on-cell with sources because of the localized mode resonance, which is helpful for subwavelength imaging.

The process of the proposed method for far-field subwavelength imaging is as follows: (1) The metalens is installed near the target to be imaged and receivers are installed in the far field. (2) The switchable SRR cells are turned on one after another, and the radiation signals are noted by the receivers. The remarkable fact regarding this system is that only one cell is turned on at given time. (3) An image of the target can be obtained, and the colour depth of each pixel depends on the maximum value in the band of 3–4 GHz of the normalized summation of the electric field intensities (NE) at the receivers when the corresponding SRR is on.

**Figure 3:**
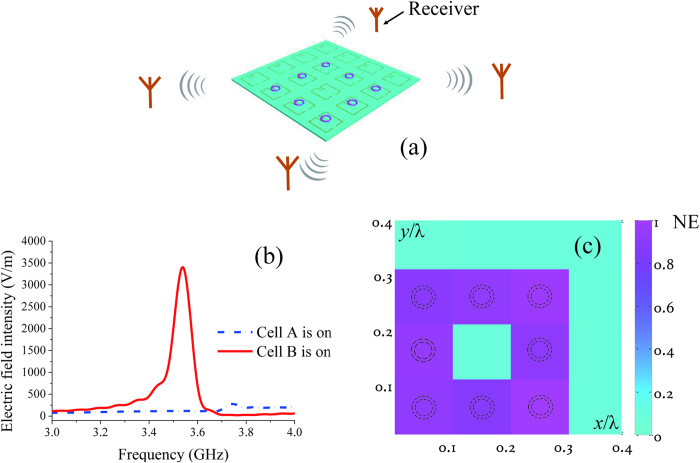
Far-field subwavelength imaging in free space. (**a**) Schematic view of the subwavelength source target and the distribution of the receivers. Four receivers in different directions are 5λ away from the SRR metalens, where λ is the wavelength at 3.55 GHz in free space. (**b**) The summation of the electric field intensities of the four receivers when different cells are turned on. (**c**) The image of the subwavelength squared target. The dotted circles are the edge profiles of the small loop sources composing the square ring target to be imaged.

**Figure 4:**
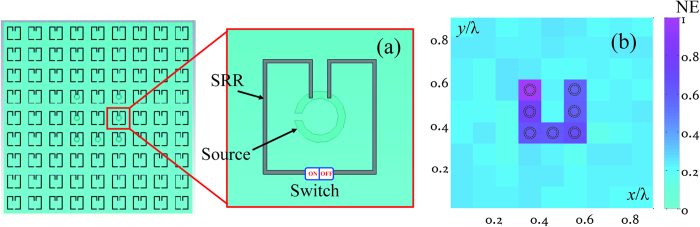
The experimental far-field image for a U-shaped target. (**a**) Structure of the experimental target. The 9 × 9 SRR cells and the U-shaped target composed of seven small loops are printed on the top and bottom of the substrate, respectively. (**b**) An image of the subwavelength target. The dotted circles are the edge profiles of the small loop sources composing the U-shaped target to be imaged.

The subwavelength squared target, which is composed of eight small loops in free space, is imaged as shown in Fig. 3. Figure 3(a) shows a schematic view of the subwavelength source target and the distribution of the receivers. Four receivers in different directions are 5λ away from the SRR metalens, where λ is the wavelength at 3.55 GHz in free space. Figure 3(b) shows the summation of the electric field intensities at the four receivers when different cells are turned on. When the cell without a source above it is turned on, the intensity at 3.55 GHz is low. When the cell with a source above it is turned on, the intensity at 3.55 GHz is much higher than at other frequencies. Figure 2 supports this phenomenon. Figure 3(c) is the image of the squared target obtained by simulation of the proposed method. In the image, the colour depth of each pixel depends on the NE of the receivers when the corresponding SRR is on. Figure 4 is an experimental example of the far-field imaging for a U-shaped target. The profile of the target is clear, emphasizing the effectiveness of the proposed method.

### Subwavelength imaging in complex environments

**Figure 5:**
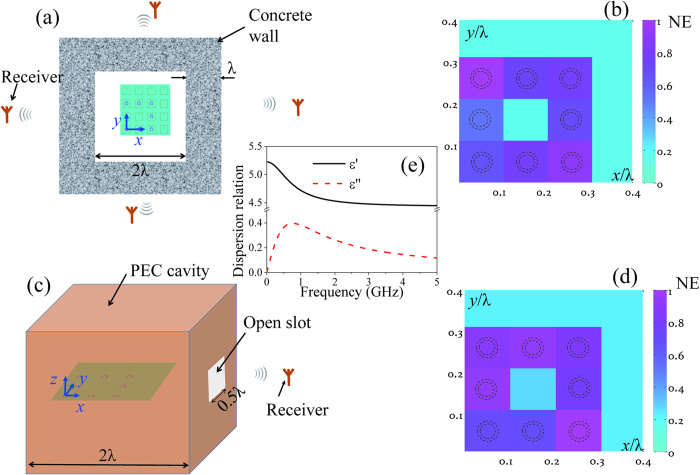
Two examples of far-field subwavelength imaging in complex environments. (**a**) Top view of the target above the metalens and the distribution of the receivers. The target is surrounded by concrete walls. (**c**) Schematic view of the target above the metalens and the distribution of the receiver. The target is located in a PEC cavity with an open slot. Images of the subwavelength targets in (**a**) and (**c**) are shown in (**b**) and (**d**), respectively. The dotted circles are the edge profiles of the small loop sources composing the square ring target to be imaged. The dispersion relation of the concrete in (**a**) is shown in (**e**).

Obtaining the Green’s function between the targets and receivers is generally necessary and important to reconstruct the target image in the far field at microwave frequencies. However, the Green’s function is difficult and time-consuming to obtain in a complex environment. Additionally, when the environment changes even a small amount, the Green’s function must be remeasured due to the environmental sensitivity of the subwavelength image. As the image of the proposed method depends on the resonant frequency of the on-SRR in the metalens, the Green’s function is not required in the imaging process. Therefore, this method is convenient and environmentally adaptable. Figure 5 shows two examples of far-field subwavelength imaging in complex environments. Figure 5(a) shows the top view of the target above the metalens and the distribution of the receivers. The target is surrounded by concrete walls. Four receivers in different directions are 5λ away from the SRR metalens, where λ is the wavelength at 3.55 GHz in the free space. Figure 5(c) shows a schematic view of the target above the metalens that is located in a perfect electric conductor (PEC) cavity with an open slot. One receiver in the direction of the open slot is 5λ away from the SRR metalens. The targets to be imaged in the two examples are the structures shown in Fig. 2(a). The images of the subwavelength targets in Fig. 5(a,c) are shown in Fig. 5(b,d), respectively. The images of the squared target surrounded by concrete walls and in a PEC cavity are both clear and agree with the structures shown in Fig. 2(a). The two examples demonstrate that the proposed far-field imaging method with near-field resonant metalens scanning can effectively image subwavelength targets in complex environments without using the Green’s function.

## Discussion

The proposed imaging method includes two key points: (1) the metalens cell with localized mode resonance can convert evanescent waves into propagating waves and can limit the surface field within a small scope, (2) the near-field scanning is realized by switching the SRR cell in this study. The near-field scanning indicates that the position in the space is coded as different points on the time axis. Therefore, the subwavelength images of targets can be obtained by comparing the spectrum performances of signals received at different times. The first key point determines the minimum resolution. In this study, the size of the switchable SRR cells is approximately 0.1λ × 0.1λ. Therefore, the size of the image pixel is also 0.1λ × 0.1λ. The second key point determines the imaging velocity because the date analysis process is simple and timesaving. That is to say the image can be obtained as soon as the plane to be imaged is scanned over. In this study, the near-field scanning is realized by changing the switch status of each SRR cell, producing an electrical or an optical scanning process when the switch is a micro-electromechanical system (MEMS) or a photoconductive semiconductor switch (PCSS). Moreover, this process can also be completed using mechanical scanning. Mechanical scanning is independent of a switchable metalens and only requires an SRR cell as the near-field scanning probe. In contrast, electrical and optical scanning methods with a switchable metalens are faster than mechanical scanning.

In conclusion, the proposed far-field imaging method with near-field resonant metalens scanning can effectively image for the subwavelength targets without using the Green’s function. This simple method can be used for subwavelength imaging, detection, and electromagnetic monitoring in both free space and complex environments.

## Methods

### Setting the simulation parameters

All simulated results are obtained using the CST Microwave Studio and all images are plotted using the MATLAB. The receivers in Figs 3 and 5 are the electric field probes. The dispersion relationship of the concrete is shown in Fig. 5(e). The other parameters of the concrete are as follows: the thermal conductivity is 1.7 W/K/m, material density is 2400 kg/m^3^, and heat capacity is 0.8 kJ/K/kg.

### Process of the experiment

The structure shown in Fig. 4(a) is fabricated by FR-4 substrate with a thickness of 0.5 mm, a relative dielectric constant of 4.6, and a loss tangent of 0.002. In total, 9 × 9 SRR cells and 7 small loops are printed on the top and bottom sides, respectively. Each SRR cell has a 1-mm slot in the middle of the metal split ring in which the switch should be installed. In the experiment, we use small copper sheets instead of MEMS switches to turn the SRR on. The 7 small loops compose a U-shaped target, and each loop is soldered to a 50-Ω coaxial cable with an SMA (Sub Miniature version A) connector. The 7 loops are excited at identical times through a power divider with the operating frequency band of 2–8 GHz. The vector network analyser (VNA, Agilent E8361A) is used for the experimental far-field imaging of the U-shaped target. The power divider is connected to port 1 of the analyser and a horn antenna with the operating frequency band of 0.2-23 GHz is connected to port 2. The distance between the U-shaped target and the horn antenna (receiver) is 400 mm (approximately 5λ to 3.55 GHz). The SRR cells are turned on one after another and the S_21_ parameter of each status between port 1 and port 2 is noted. After changing the direction of the receiver, the S_21_ of each status is noted again. This measuring process is repeated until four S_21_ of each status are obtained. After that, the image of the subwavelength target is plotted in MATLAB. In the image, the colour depth of each pixel depends on the maximum value of NE in the 3–4 GHz band when the corresponding SRR is turned on.

## Additional Information

**How to cite this article**: Wang, R. *et al.* Far-field subwavelength imaging with near-field resonant metalens scanning at microwave frequencies. *Sci. Rep.* **5**, 11131; doi: 10.1038/srep11131 (2015).
